# Personalised Dosimetry in Radioembolisation for HCC: Impact on Clinical Outcome and on Trial Design

**DOI:** 10.3390/cancers12061557

**Published:** 2020-06-12

**Authors:** Etienne Garin, Xavier Palard, Yan Rolland

**Affiliations:** 1 Univ Rennes, Inra, Inserm, Centre de Lutte Contre le Cancer Eugène Marquis, Institut NUMECAN (Nutrition, Métabolismes et Cancer)—UMR_A 1341, UMR_S 1241, F-35000 Rennes, France; 2 Centre de lutte contre le Cancer Eugène Marquis, F 35042 Rennes, France; x.palard@rennes.unicancer.fr; 3 Univ Rennes, CLCC Eugène Marquis, INSERM, LTSI—UMR 1099, F-35000 Rennes, France; y.rolland@rennes.unicancer.fr

**Keywords:** dosimetry, radioembolisation, hepatocellular carcinoma

## Abstract

Selective internal radiation therapy (SIRT) of hepatocellular carcinoma (HCC) has been used for many years, usually without any specific dosimetry endpoint. Despite good clinical results in early phase studies or in cohort studies, three randomized trials in locally advanced HCC available failed to demonstrate any improvement of overall overall survival (OS) in comparison with sorafenib. In recent years, many studies have evaluated the dosimetry of SIRT using either a simulation-based dosimetry (macroaggregated albumin (MAA)-based) or a post-therapy-based one (^90^Y-based). The goal of this review is to present the dosimetry concept, tools available, its limitations, and main clinical results described for HCC patients treated with ^90^Y-loaded resin or glass microspheres. With MAA-based dosimetry, the threshold tumor doses allowing for a response were between 100 and 210 Gy for resin microspheres and between 205 and 257 Gy for glass microspheres. The significant impact of the tumor dose on OS was reported with both devices. The correlation between ^90^Y-based dosimetry and response was also reported. Regarding the safety, preliminary results are available for both products but with a larger range of normal liver doses values correlated with liver toxicities due to numerous confounding factors. Based on those results, international expert group recommendations for personalized dosimetry have been provided for both devices. The clinical impact of personalized dosimetry has been recently confirmed in a multicenter randomized study demonstrating a doubling of the response rate and an OS of 150% while using personalized dosimetry. Even if technical dosimetry improvements are still under investigation, the use of personalized dosimetry has to be generalized for both clinical practice and trial design.

## 1. Introduction.

Selective internal radiation therapy (SIRT) is used form many years for the treatment of nonoperable hepatocellular carcinoma (HCC) patients with locally advanced disease. ^90^Y-loaded microspheres, either glass microspheres (TheraSphere^®^, Boston Scientic Corporation, Marlborough, MA, USA) or resin microspheres (SIR-Sphere^®^, Sirtex Medical Limited Australia, Sidney, Australia), are the most common products used. SIRT is always preceded by a diagnostic liver angiography combined with intra-arterial injection, at the treatment position, of ^99m^Tc macroaggregated albumin (MAA) to perform a liver perfusion scintigraphy (MAA scan). The objectives of those tools are to perform an arterial mapping and to identify patients with contra-indication to receive SIRT (patients with high lung shunting providing an absorbed dose of more than 30 Gy to lungs or at risk of gastro-intestinal shunting).

Many promising results based on cohort studies and phase II studies have been reported [1,2,3,4,5,6], with response rates (RRs) based on mRECIST or EASL criteria between 50% and 86% [1,5] and the overall survival (OS) reaching more than 20 months for patients with portal vein thrombosis (PVT) [6]. SIRT is recommended in several guidelines as the National Comprehensive Cancer Network (NCCN) guidelines for HCC [7] and in the European Society for Medical Oncology (ESMO) guidelines [8] in patients with early, intermediate-stage, or advanced stages of HCC based on the Barcelona Clinic of Liver Cancer (BCLC). However, all phase 3 trials comparing SIRT with sorafenib have been failed to demonstrate any OS improvement using SIRT in comparison with that using sorafenib [9,10,11]. Several concerns, which could explain the negativity of those studies, have been raised particularly in the absence of dosimetry endpoints, despite the fact that SIRT is a radiation oncology approach, where radiobiological rules apply [12]. For radio-induced deterministic effects, a threshold absorbed dose is mandatory to observe an effect, and the higher the absorbed dose is above this threshold, the more severe the effect is. This phenomenon exists, until the maximal effect is achieved (complete histological necrosis). Treatment planning is usually based either on an activity of ^90^Y in GBq to administer related to the body surface area with SIR-Sphere^®^ [13] or on an absorbed dose delivered to the liver (80 to 150 Gy) with TheraSphere^®^ [14]. However, in reality, SIRT planning should be based on a tumoricidal tumor dose (TD) necessary to induce a tumor response and on a normal liver dose (NLD) not to exceed to avoid liver decompensation.

For liver SIRT, two dosimetry approaches are available, i.e., a simulation dosimetry based on MAA quantification prior to a treatment, allowing for a potential dosimetry personalization, or a direct ^90^Y quantification after a treatment, assumed to be more accurate but not allowing for personalized dosimetry.

The purpose of this article is to recall the dosimetry concept and limitations, to review the available clinical data about MAA-based dosimetry, ^90^Y dosimetry, the development of personalized dosimetry, and potential impact on trial design for HCC patients.

## 2. Dosimetry Concept and Limitations

### 2.1. Evaluation of the Physically Absorbed Dose

The physical definition of an absorbed dose “D” is an energy “E” deposition (Joule) in a mass “M” (kg):

D_(Gy)_ = E_(J)_/M, (1)

where D is expressed in Gray (Gy) with 1 Gy = 1 J/kg.

Several dosimetry algorithms providing the physically absorbed dose D in SIRT are described including the medical internal radiation dose (MIRD) approach, Monte Carlo simulation, and kernel point evaluation [15]. The MIRD approach is actually the most widely approach used in clinical practice [15,16].

### 2.2. The MIRD Approach

The MIRD approach assumes a homogeneous distribution of the energy deposition.

The energy deposition for 1 GBq of ^90^Y in a mass of 1 kg is 50 Gy, regardless of the nature of the mass.

The absorbed dose “D” in a volume of interest (VOI) of mass “M” (in kg) and containing an activity “A” of ^90^Y (in GBq) is calculated using the following simplified MIRD equation:

D_(Gy)_ = A _(GBq)_ × 50/M. (2)

**Figure 1:**
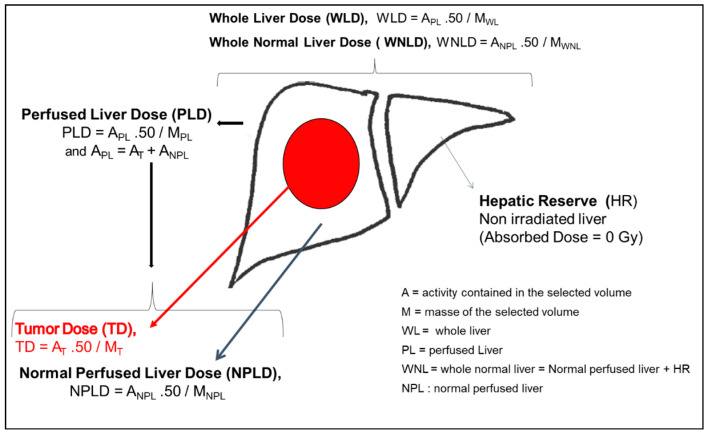
Example of dosimetry parameters for a treatment. A = activity contained in the selected volume (ex: A_PL_ = Activity contained in the Perfused Liver); M= Mass of the selected volume; HR = Hepatic Reserve; NPL = Normal Perfused Liver; NPLD = Normal Perfused Liver Dose; PL= Perfused liver; PLD = Perfused Liver Dose; T = Tumor; TD = Tumor Dose; WL = Whole Liver; WLD = Whole Liver Dose; WNL= Whole Normal Liver; WNPLD = Whole Normal Perfused Liver Dose.

Doses can be calculated for different VOIs including lungs, the liver, the tumor, the perfused liver and the normal perfused liver (Figure 1).

Regarding the liver, the unicompartment model assumes a homogeneous distribution between the tumor and the normal liver, and only the perfused liver dose is evaluated. This approach is a standard approach used in the instruction for the use of glass microspheres.

In the multicompartment approach, a TD and an NLD are also evaluated.

To evaluate an absorbed dose in clinical practice, only two parameters are required, i.e., the volume of the structure of interest (as the mass is proportional to the volume) and the activity of ^90^Y contained in this volume.

Taking into account the fact that there is no sphere redistribution after initial embolization in the microvascularization and no biodegradation, it is assumed that microsphere deposition is only in the liver (including tumors) and eventually lungs (if arterio-venous shunt is present in the tumor), and then the activity of ^90^Y with a VOI depends on the activity of ^90^Y injected to the patient (A_I_) and the fraction of ^90^Y uptake in this VOI.

^90^Y uptake can be evaluated before the treatment, during the treatment simulation, using ^99m^Tc macro-aggregated albumin (MAA) SPECT/CT as a surrogate of microsphere distribution or after the treatment, directly with ^90^Y SPECT/CT or more accurately with ^90^Y PET/CT, and doses are calculated as following.

For lungs, the mass is, by approximation, assumed to be constant (1 kg), then the dose in lungs are written as:

Lung D_(Gy)_ = A_I(GBq)_ × LSF × 50, (3)

where LSF is the lung shunt fraction and is described as the total lung uptake/(the total lung uptake + the total liver uptake).

For the liver and tumors, assuming that:

M (kg) = Volume (L) × 1.03(4)

Then, the doses in the liver and tumors are expressed as:

Perfused liver dose (PLD) = A_I(GBq)_ × (1 − LSF) × 50/(perfused liver volume × 1.03), (5)

Tumor dose (TD) = A_I(GBq)_ × (1 − LSF) × TUR. 50/(tumor volume × 1.03), (6)

where TUR is the tumor uptake ratio and described as the total tumor uptake divided by the total perfused liver uptake.

Normal perfused liver dose (NPLD) is obtained by the subtraction (a volume activities) of the tumors to the perfused liver.

Doses can also be extrapolated to the whole liver and the whole normal liver (WNL) (Figure 1).

Doses can be calculated as a mean dose of a VOI, which is the simplest approach.

Doses can also be calculated at the voxel level. Voxel dosimetry allows for the generation of a dose volume histogram (DVH), and then a mixed parameter based on the dose and the volume can be generated. For example, the D70 is the minimum dose applied to 70% of a VOI and currently used with external beam radiotherapy [17].

Tumor control probability (TCP) curves (providing the probability of control for a tumor-absorbed dose) as nontumor complication probability (NTCP) curves (probability of complication for an NPLD) can be generated.

Several kinds of dosimetry software are now available for clinical practice.

### 2.3. Limitations of the MIRD Approach

Radiobiological effects depend not only on physically absorbed doses but also on dose rates and the heterogeneity of the dose distribution. One main limitation of the MIRD approach is that the heterogeneity of the dose distribution is not taken into account. It does not take into account the difference of the heterogeneity of the dose distribution observed between both products related to a different specific activity (50 Bq by resin spheres and 2500 Bq by glass spheres at a calibration time) and then different numbers of spheres injected (factor 50) for the same injected activity of ^90^Y.

Then, despite the fact that the MIRD equation is currently used for glass and resin microspheres, for the same absorbed dose, radiobiological effects are different. This point has been demonstrated at the level of the normal parenchyma at the lobule scale by a simulation study provide by Warland et al. [18], where for a whole liver irradiation the doses delivered to the liver providing 50% of toxicity were 40 Gy for resin microspheres and slightly higher than 60 Gy for glass microspheres. The clinical data also support this difference of radiobiology between both products. For example, the threshold tumor doses (TTDs) reported to be related with the response were between 100 and 120 Gy for resin microspheres [19,20] and between 205 and 257 Gy for glass microspheres [15,21,22].

Voxel dosimetry and dose volume histograms (DVH) evaluation takes into account, at the voxel level, the heterogeneity of the dose distribution, which can be observed in a large volume; however, the microscopic heterogeneity of the dose distribution is not taken into account.

To minimize the difference of radiobiological effects that can be seen for the same physically absorbed dose (as calculated with MIRD) between different types of irradiation and irradiated tissues, the biologically effective dose (BED) can be implemented, which is based on the correction of a physically absorbed dose with different radiobiological parameters [15]. The use of BED has been described with SIRT, but the difficulty is that the corrected radiobiological parameters used are those defined in external beam radiotherapy with a different irradiation configuration and their accuracy with SIRT is a matter of debate.

### 2.4. Confounding Factors of Dosimetry Evaluation

Many confounding factors with a direct impact on dose evaluation and correlation with outcomes have been described including the dosimetry performed (MAA-based or ^90^Y-based), the tumor size (with a risk of dose underestimation for small lesions due to partial volume effects), tumor histology and vascularization, prior therapy, concomitant/or subsequent therapy, response, or toxicity criteria used [16,23].

Two important technical issues have also to be highlighted: segmentation and angiographic requirement for a simulation-based dosimetry (MAA-based) [16,23,24].

#### 2.4.1. Segmentation

For the segmentation of a VOI (and therefore for the volumes evaluation), two approaches are available [16,23].

The first one is diagnostic imaging using CT, MRI, or CBCT. This kind of imaging is required to be coregistered with SPECT/CT or PET to evaluate the count number in a VOI. In this situation, only the counts within anatomically delineated VOIs are taken into consideration for the dose calculation of this VOI. The advantage of this approach is the achievement of the most accurate and reproducible volume definition. However, in case of coregistration error, a significant underestimation of the absorbed dose in the VOI can be observed due to an underestimation of the counts in the VOI.

The second approach available is based on a full SPECT/CT (or PET/CT) segmentation, which is semi-automated and thresholding-based, and it has been previously validated by a phantom study, where the mean error in the volume measurement was lower than 7% with good reproducibility [24]. In this situation, the segmentation provides both the volume and the counts included in this volume, and then a coregistration of the SPECT or PET with a diagnostic imaging is required. However, in some complex cases, the thresholding may be difficult to perform.

The impact of the segmentation approach used on dosimetry evaluation can be high as seen in one study evaluating ^90^Y-PET dosimetry based on CT segmentation, which failed to identify any dose–response relationship on HCC [25] and in others studies using a SPECT/CT segmentation for MAA dosimetry, where a dose–response relationship has been demonstrated [15,19,20,21,22].

#### 2.4.2. Specific Angiographic Requirements for a Simulation-Based Dosimetry

We have to consider the simulation-based dosimetry as a global approach including angiographic considerations, and this approach cannot be limited to an accurate quantification of the surrogate itself.

In this situation, the blood flow must be similar between the simulation angiography and the treatment itself. Several technical issues have been described: the spasm occurrence [16,23,26], the proximity of arterial bifurcation [27], the speed of surrogate injection [16], and the catheter repositioning [25,28]. Several recommendations have been drawn to control the blood blow and improve the accuracy of the simulation-based dosimetry [16,23,29]:

- Limiting the risk of spasm, whenever technically possible, avoiding coil embolization and favoring the use of floppy catheter;
- Taking care of bifurcation proximity, more than 1 cm from the catheter tip, whenever technically possible;
- Slow injection of a microsphere surrogate (over 20 to 30 s);
- Injection of a surrogate and ^90^Y microspheres exactly at the same position, including the catheter tip orientation in the arterial tree.

## 3. MAA as a Surrogate of Microspheres

MAA scanning was initially developed for lung shunt evaluation [30].

MAA particles sizes are, in 90% of cases, between 10 and 40 μm, which is in the same range as that of resin (20–60 µm) or glass (20–30 µm) microspheres. However, 1 to 2% of MAA particles are less than 15 μm, and it is recognized that MAA can lead to an overestimation of lung shunting, which has been definitely demonstrated in a study evaluating lung shunt either with MAA or with holmium microsphere [31]. Several studies were focused on the evaluation of the correlation between MAA and ^90^Y uptake and dosimetry. Several studies found a poor correlation between MAA and ^90^Y uptake or quantification in tumors and normal liver tissues. They were mainly carried out in patients with metastatic disease using either resin microspheres [28,32,33,34], which were biased by several technological issues as catheter repositioning [32,33,34] or the absence of spasm evaluation [25,28,32,33,34]. On the other hand, more and more studies have confirmed MAA as a microsphere surrogate, at least in HCC, regardless of microspheres used, even if for some individual cases discrepancies are present [27,35,36,37,38,39,40].

## 4. MAA-Based Dosimetry, Response, Overall Survival (OS) and Hypetrophy

Instead of comparing MAA dosimetry and ^90^Y dosimetry, several retrospective studies have evaluated MAA dosimetry and outcomes and demonstrated a clear dose/response relationship (Table 1) and an impact of TD on OS (Table 2).

### 4.1. Resin Microspheres

Three major studies are available for resin microspheres. The first one published more than 25 years ago by Lau et al. [19] identified a TTD of 120 Gy with an RR of 85.7% for a TD of ≥120 Gy versus 12.5% for a TD of <120 Gy (*p* = 0.005). Median OSs were 55.9 weeks for a TD of ≥120 Gy and 26.6 weeks for a TD of <120 Gy (*p* = 0.005) [19]. The results of the second one were presented by Herman et al. during EASL 2018 Congress [20]. Over 121 evaluable patients regarding the dosimetry of a SARAH trial, a TTD of 100 Gy was identified with a disease control rate of 65.6% for a TD of ≥100 Gy or that of 34.4% for a TD of <120 Gy (*p* = 0.0047) [20]. Median OSs were 14.1 months for a TD of ≥100 Gy and 6.1 months with a relative death risk of 2.7 for a TD of <100 Gy (*p* < 10^−3^) [20]. Finally, Kao et al. [43] evaluated retrospectively ^90^Y SPECT/CT dosimetry (MIRD) on 10 patients. All patients received a TD of >91 Gy and were responders based on RECIST criteria.

### 4.2. Glass Microspheres

**Figure 2:**
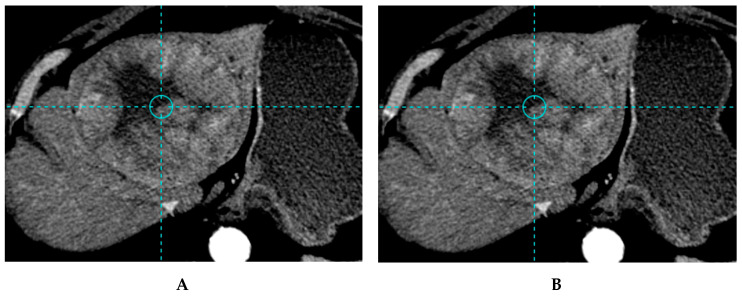
Example of a patient treated with the standard dosimetry approach, with a lesion under-treated (tumor dose below the 205 Gy threshold) (**A**) Baseline CT image of bilobar HCC with a 10.7 cm lesion of the left lobe and a 5.2 cm lesion of segment V in a 70-year-old patient with good performance status (ECOG 0) and liver functions (Child Pugh A5). The patients received a treatment of the left lobe with 1.93 GBq of ^90^Y glass microspheres using the standard dosimetry approach delivering 140 Gy to the large lesion and 117 to the left lobe. On the CT image, 3 months after SIRT, stable disease was observed (**B**). Overall survival of this patient was 8.7 months.

**Figure 3:**
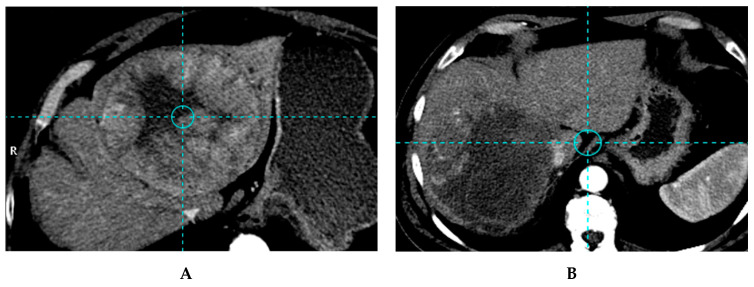
Example of a patient treated with a personalised dosimetry approach, with a lesion accurately treated (tumor dose higher than the 205 Gy threshold). (**A**) Based CT image of 15 cm unifocal right HCC with the invasion of the veina cava in a 64-year-old patient with good performance status (ECOG 0) and liver functions (Child Pugh A5). The patient received a treatment of the right lobe with 7.06 GBq of ^90^Y glass microspheres using the personalized dosimetry approach delivering 294 Gy to the large lesion and 173 Gy to the right lobe. CT image, 3 months after SIRT, partial response of the huge lesion was obtained using EASL criteria, as no recurrence on the left lobe and no extra hepatic spread (**B**). The patient was still in good performance status (ECOG 0) and liver functions (Child Pugh A5) and a right hepatectomy was performed with veina cave reconstruction. At last follow-up evaluation, 22 months after SIRT, the patient was still in complete response.

With glass microspheres, four major studies are available. In the first study (52 patients), a TTD of 257 Gy was identified with a sensitivity of 85% and a specificity of 70% in response prediction [15]. The impact of TD on OS was not provided. In the second study (36 patients), a TTD of 205 Gy was identified with a sensitivity of 100% and an accuracy of 91% for response prediction [21], (Figure 2 and Figure 3). The median OS was significantly improved for patients, who received a TD of ≥205 Gy (18 months; 95% CI: 11–NR), compared to those receiving a TD of <205 Gy (9 months; 95% CI: 2–31) (*p* = 0.032) [21]. This TTD of 205 Gy was subsequently confirmed in a study on 85 patients and 132 evaluable lesions [22]. The RRs were 89.7% for a TD of ≥205 Gy and 9.1% for a TD of <205 Gy (*p* < 10^−7^) [22]. The median OSs were 21 months for a TD of ≥205 Gy and 6.5 months for a TD of <205 Gy, with a relative risk of death of 2.35 for a TD of <205 Gy (*p* = 0.0072) in an unselected population [22]. In HCC patients, the impact of TD on median OS was even higher with a relative risk of death of 6.99 for a TD of <205 Gy (*p* = 0.0025) [22]. The fourth study brings interesting results with a TTD depending on HCC differentiation, with a TTD of 152 Gy, 174Gy and 262 Gy for well, moderately and poorly differentiated lesions, respectively, with a 89.2% sensitivity and a 88% specificity [40].

### 4.3. MAA Dosimetry and Hypertrophy

Only one study has evaluated the potential impact of dosimetry and future remnant liver (FLR) hypertrophy, and was based on MAA evaluation [46]. In this retrospective cohort of 73 HCC patients treated with glass microspheres, the mean FLR hypertrophy was 35.4 ± 40.4%. An FLR hypertrophy rate of ≥10% was significantly more frequent for patients with NPL Ds of ≥88 Gy, i.e., 92.2%, compared to 65.7% for NPLDs of <88 Gy (*p* = 0.032) [46]. An FLR hypertrophy rate of ≥10% was also significantly more frequent for patients with a TD of ≥205 Gy and a tumor volume (VT) of ≥100 cm^3^ in patients with an initial FRL rate of <50%. Finally, an FLR hypertrophy rate of ≥10% was seen in 83.9% of the patients with either an NPL D of ≥88 Gy or a TD of ≥205 Gy for tumors larger than 100 cm^3^ (85% of the cases), and an FLR hypertrophy rate of ≥10% was only found in only 54.5% (*p* = 0.0265) of patients with none of those parameters [46].

For the first time, this study suggests that it is possible to stimulate FRL hypertrophy using personalized dosimetry, increasing the dose to the NPL or to large tumors.

## 5. ^90^Y-Based Dosimetry, Response, and OS

A couple of studies have evaluated ^90^Y dosimetry.

### 5.1. Resin Microspheres

The first study based on ^90^Y SPECT/CT dosimetry (Monte Carlo dose voxel kernel and BED) was published by Strigari et al. [44]. The authors evaluated the TCP in a cohort of 73 HCC patients and found a TCP of 73%, based on EASL response criteria, for a TD of 110 Gy. The tumor control was based on EASL response criteria including only complete and partial responses.

Allimant et al. [45] evaluated ^90^Y PET dosimetry using the MIRD approach and the area under the dose–volume histogram as a TD parameter in 37 patients and 42 procedures [45]. The area under the tumor dose volume histograms (AUDVHs) of ≥61 Gy were predictive of tumor control with a 76.5% sensitivity and a 75% specificity. Tumor control was defined as the sum of complete response, partial response and stable disease, evaluated with mRECIST criteria at six months. They also evaluated the tumor coverage as a predictor of response and found that both AUDVHs and tumor coverage were associated with tumor control using multivariate analysis.

The difference of the values of the TD parameter predicting tumor control identified in those studies (from 61 to 110 Gy) typically illustrates the difference we can observe due to different dosimetry approaches (Monte Carlo versus MIRD) and different criteria of response/control we can also use.

### 5.2. Glass Microspheres

Chan et al. [41] evaluated the TD using ^90^Y PET and the Monte Carlo approach prospectively in 27 patients and 38 tumors. Response evaluation was based on mRECIST. A close dose/response relation was demonstrated with a median TD and a median D70 of 225.1 and 140.0 Gy, respectively, for responders and with those of 82.7 and 24 Gy, respectively, for nonresponders (*p* < 0.01). All nonreponders had a TD of <200 Gy.

Kappadath et al. [42] evaluated retrospectively the tumor mean dose based on ^90^Y SPECT/CT and the Monte carlo approach in 34 patients. A mean TD of 160 was predictive of a 50% probability of response evaluated using mRECIST criteria, and responders complete and partial responses were considered.

No results were provided in those four studies regarding TD parameters potential impact on OS.

## 6. Dosimetry and Liver Toxicity

The maximal liver-tolerated dose is more complex to define, as several confounding factors must be taken into account, such as toxicity definition (including grade and reversibility), treatment line, underlying liver disease and severity, and hepatic reserve [16,23].

A specific syndrome was described by Sangro et al. The Radioembolisation-induced liver disease (REILD) was defined by the occurrence during the first 2 months after SIRT with a bilirubin concentration rise over 51 µmol/L and/or ascites, in the absence of tumor progression or bile duct dilatation [47].

It is possible to evaluate the NPLD and the hepatic reserve (nonirradiated liver) separately and to include the hepatic reserve in the dose calculation, calculating the whole normal liver dose (WNLD) as proposed by Chiesa et al. [48]. (Figure 1).

### 6.1. Resin Microspheres

Strigari et al. [44] evaluated the NPLD (^90^Y SPECT/CT, Monte Carlo dose voxel kernel and BED) in 73 patients. The median NPLD was 36 Gy (range: 6–78 Gy), and Grades of ≥2 (G2), ≥3 (G3), and ≥4 (G4) liver toxicities were observed in 32% (23/73), 21% (15), and 11% (8) of patients, respectively. An NPLD of 52 Gy (95% CI: 44–61 Gy) was identified to predict a 50% probability of ≥G2 liver toxicity in this patient group treated by a whole liver approach (absence of the hepatic reserve).

Allimant et al. [45] evaluated the AUDVH as a TD parameter in 38 patients (^90^Y PET dosimetry, MIRD approach). The AUDVH for the NPL was significantly higher for patients with liver toxicity than for those without liver toxicity (78.91 versus 53.84 Gy; *p* = 0.04). The liver toxicity was defined as radioembolisation induced liver disease (REILD) as described initially by Sangro et al. [48]. Studies are summarized in Table 3.

### 6.2. Glass Microspheres

In the initial development of glass microspheres, only unicompartment dosimetry is used, and is has been demonstrated that the tolerance of a whole liver dose up to 140 Gy is acceptable [49,50].

Using the multicompartment approach, Chiesa et al. [48] evaluated the WNLD, including irradiated and nonirradiated parenchyma (MAA-based dosimetry, MIRD approach) in a cohort of 52 patients. They demonstrated that a WNLD of 75 Gy can induce a 15% probability of liver decompensation, irrespective of its severity and eventual reversibility. Studies are summarized in Table 3.

NPLD was also evaluated (MAA-based dosimetry, MIRD approach) in a cohort comprising 71 patients, with 94.4% of them exhibiting a Child-Pugh A score [5]. The NPLD and hepatic reserve alone did not correlate with severe clinical permanent liver toxicity (Common Terminology Criteria for Adverse Events V3, G ≥ 3). Only the association of a NPLD of >100 Gy with a hepatic reserve of <30% correlated with severe permanent liver toxicity upon univariate analysis (*p* = 0.032) [5]. However, in a more recent study (MAA-based dosimetry, MIRD approach), a significant difference of the NPLD between patients with liver-related toxicities and those without liver-related toxicities (104.7 ± 33.1 versus 79.5 ± 29.1 Gy; *p* = 0.0283) was shown [22].

NPLD was evaluated with ^90^Y PET in one study (Monte Carlo approach) including 34 patients (27 with HCC and 7 with liver metastasis), [51]. An NPLD threshold of 54 Gy was predictive of liver toxicity probability of more than 50%. In this study, toxicities of grade 2 were taken into account including laboratory test toxicities.

Finally, for patients with portal vein thrombosis (PVT), another parameter beyond the liver dose has a major impact on safety, i.e., the PVT targeting [5,6,22]. Indeed, in two studies, the NLD evaluated either alone or associated with a low hepatic reserve was not associated with liver toxicity for PVT patients. In this situation, the only parameter strongly associated with liver toxicity was the absence of MAA PVT targeting [6,22].

One limitation of the studies evaluating NLD and safety is the fact that the dosimetry evaluation was performed for the patients with several SIRT treatments only for the first SIRT treatment. In this situation, the cumulative liver dose was not evaluated.

## 7. Personalized Dosimetry

Personalized dosimetry can be developed only using MAA-based dosimetry, which is the only evaluation available prior to ^90^Y-loaded microspheres injection. The results of four studies are available only for glass microspheres.

In one retrospective study, the main dosimetry endpoint was to provide the supposed maximal tolerated dose of the WNL, regardless of the TD [52]. One-hundred and twenty HCC patients with PVT were treated using this concept with an initial limit of 40 Gy injected to the WNL for the first 18 patients and with an initial limit of 70 Gy injected to the WNL for the remaining patients. The median OS reported was 14.1 months (95% CI: 10.7–17.5) compared favorably with median OSs of 10.4 (95% CI: 7.2–16.6) and 10 months (95% CI: 7.7–10.9) reported in cohorts of PVT patients treated without personalized dosimetry [1,4].

In two others retrospective studies, the dosimetry endpoints based on MAA SPECT/CT were to target at least 205 Gy to the tumor while limiting the NPLD below 120 Gy [5,6]. The first one reported the results on the unselected 71 HCC patients [5]. For the global cohort, the RR was 78.8%. It was significantly improved in the 51 patients treated with personalized dosimetry in comparison with the 20 patients, who received standard dosimetry (86% versus 55%; *p* = 0.001). Using a TDD of 205 Gy, the false positive rates (TD ≥ 205 Gy and no response) were 15.4% for TDs between 205 and 275 Gy, 8.1% for a TD of >275 Gy and 0% for a TD of >350 Gy, illustrating the radiobiological rule, i.e., the higher the dose above the TTD, the higher the damage is. No difference in clinically relevant liver toxicity of grade of ≥3 was reported in the 17 patients, who received a treatment intensification (injected lobe dose of >150 Gy), i.e., observed at a 5.8% frequency, in comparison with a 9.2% frequency reported for the patients not intensified. The median OSs were 23.2 months for a TD of ≥205 Gy and 13 months for a TD of <205 Gy, but the difference was not statistically significant. The second study was focused on 41 PVT patients [6]. The median OS of the global cohort was 18 months. Patients were classified as good candidates to receive SIRT if the TD was ≥205 Gy and if the MAA PVT targeting was good and poor candidates to receive SIRT if either the TD was <205 Gy or the MAA PVT targeting was poor. The median OSs were 20.2 months (95% CI: 12–25.1) for good candidates and only 3 months (95% CI: 3–3.7) for poor candidates (*p* < 0.001). Poor candidates based on the treatment simulation (TD and MAA targeting) representing 12.2% of the population in this study were 37% of the patients received a treatment intensification (more than 150 Gy for the lobe). As interesting results, five patients (12.2%) were downstaged to surgery with a complete portal vein revascularization.

The results of a multicentre randomized phase 2 study comparing personalized dosimetry and standard dosimetry with glass microspheres in patients with a least one lesion larger than 7 cm were recently communicated [53]. In this study, 60 patients were randomized to receive standard dosimetry (perfused liver dose of 120 ± 20 Gy) or personalized dosimetry (targeting more than 205 Gy to the tumor). The superiority of personalized dosimetry with an RR of 71.4% in the personalized dosimetry arm over in the standard dosimetry arm was demonstrated (median OS of 26.7 m (95% CI: 11.7–NR) for personalized dosimetry versus 10.6 m (95% CI: 6–16.8) for standard dosimetry; *p* = 0.0096).

## 8. Recommendations for Personalized Dosimetry

Two dosimetry recommendation papers have been published with one for resin microspheres and the other for glass microspheres [29,54].

For resin microspheres [54], it has been recommended to deliver 120 Gy to HCC lesion. The recommendation to reduce the injection of ^90^Y-loaded microspheres was based on underlying liver cirrhosis and NPLD. For patients based on underlying liver cirrhosis, the NPLD was less than 50 Gy. For patients without underlying liver cirrhosis, the NPLD was less than 70 Gy.

For glass microspheres, recommendations were drawn for four different clinical scenarios and were based on published clinical data with different dosimetry endpoints depending on scenarios [29]. The main objective of the recommendations was to unify users behind the standardized dosimetry methodology that is simple and reproducible.

Two scenarios with curative intent are described, i.e., the radiation segmentectomy and the radiation lobectomy scenarios [29]. For those two scenarios, recommendations are still based on an absorbed dose to deliver to the perfused volume (unicompartment dosimetry) with at least 190 Gy to ≤2 segments (with contemporary data supporting the use of 250–300 Gy) for radiation segmentectomy and 140–150 Gy for radiation lobectomy.

Two palliative scenarios were described, but in reality, they are very close regarding dosimetry endpoints, i.e., “multifocal unilobar/bilobar HCC without macrovascular invasion” and “HCC with macrovascular invasion” [29]. In both scenarios, it is recommended to evaluate the doses for the normal parenchyma and the TD (multicompartment dosimetry). Regarding the safety, it is recommended to deliver no more than 75 Gy to the whole normal parenchyma (mean dose to the treated and untreated WNL parenchyma) for Child Pugh A patients. Regarding the efficacy, it is recommended to deliver more than 200 Gy to the tumor. For PVT patients, PVT targeting has to be evaluated by MAA scanning.

## 9. Impact on Study Design

Actually, we obtained good clinical results observed with SIRT in retrospective studies and in a couple of phase 2 studies but with a negativity of three randomized phase 3 trials published.

Despite the patient selection that might be not accurate enough, two major methodological concerns have been raised, i.e., the inclusion of patients with contraindication to receive SIRT and the absence of dosimetry endpoints [12].

In all negative phase 3 trials, randomization was performed before excluding patients with high lung shunt or digestive shunt, which is an absolute contraindication mentioned in the instruction for use of the product, meaning before performing MAA scans, despite the knowledge that lung shunt occurs in more than 20% of HCC [30]. As a consequence, between 22% and 28% of the patients in the SIRT arms did not received SIRT in SARAH and SIRveNIB trials [9,10,11]. To avoid this important bias, randomisation has to be performed after the identification of contraindications, as in every trial where the primary endpoint is median OS in the intent to treat population, meaning after the MAA scan. Recent clinical data based on retrospective studies and dosimetry recommendations made by international experts support the use of personalized dosimetry. The communication of the DOSISPHERE results definitely brings the level 1 evidence that personalized dosimetry has to be used. This is a second strong argument to perform the randomization after the MAA. Patients with a TD lower than the TDD are recognized for the product used, and patients with poor PVT targeting should also be excluded.

Accurate patient selection is also a challenging point in trials on HCC patients. This is especially true with SIRT, where the treatment can be really effective and responsible for liver decompensation.

For SIRT, several criteria are well recognized as good performance status (ECOG 0 or 1) and preserved liver functions (Child Pugh A), but others are less recognized as PVT targeting and tumor targeting. Therefore, MAA-based TD has to be used.

Whether to choose a tumor load of less than 70% or less than 50% is also a matter of debate, and tumor extension (unilobar or bilobar) has to be discussed regarding the safety. In DOSISPHERE-01 study exclusion of patients, where it is not possible to spare from radiation, at least 30% of the liver volume is a good point, because it is possible to increase doses without increasing liver toxicities [53].

## 10. Conclusions

In recent years, many studies have evaluated the dosimetry of SIRT using either a simulation-based dosimetry (MAA-based) or a post-therapeutic-based one (^90^Y-based). With MAA-based dosimetry, the significant impacts of the TD on the response and the OS were reported with both devices. The correlation between ^90^Y-based dosimetry and response were also reported. The use of personalized dosimetry, based on MAA dosimetry, has been developed, and its major clinical impact on response and OS have been validated in a randomized multicenter phase II study with glass microspheres. Recommendations for personalized dosimetry raised by the international expert group have been provided for both devices. Personalized dosimetry has to be generalized in clinical practice and in trial design.

## Figures and Tables

**Table 1:** Studies with tumor dose outcome correlation in hepatocellular carcinoma (HCC) with glass micropsheres.

Author and Year of Publication	Chiesa 2011 [15]	Garin 2012 [21]	Garin 2017 [22]	Ho 2018 [40]	Chan 2018 [41]	Kappadath 2018 [42]
Nb of patients/lesions	48/65	36/58	85/132	62/na	27/38	34/53
Lesion size (cm)	5.6	7.1	7.1	na	7.3	4.1
Macroaggregated albumin (MAA)- or ^90^Y-based dosimetry	MAA-based	MAA-based	MAA-based	MAA-based	^90^Y PET	^90^Y SPECT/CT
Response evaluation	EASL	EASL	EASL	^18^FDG or ^11^C-acetate PET	mRECIST	mRECIST
Tumor dose (TD) parameter (Gy)/threshold TD (TTD)	mean TD 257 Gy	mean TD 205 Gy	mean TD 205 Gy	mean TD 152/174/262 Gy	mean TD 200 Gy	mean TD 160 Gy
RR for TD ≥ TTD vs. < TTD	85% vs. na	na	91% vs. 5.5% *p* < 10^−3^	na	84% vs. na	50% TCP
Prediction of response for TTD	se = 85% spe = 70%	se = 100% acc = 91%	se = 98.3% acc = 88.7%	se = 89.2% spe = 88%	se = 66% PPV = 100%	na
OS for TD ≥ vs. < TTD	na	18m vs. 9m *p* = 0.032	21m vs. 6.5m *p* = 0.0052	na	na	na

Nb = number; na = not available; TTD = threshold tumor dose; w = week, m = months; se = sensitivity; spe = specificity; PPV = negative predictive value; acc = accuracy; TCP = tumor control probability; HCC = hepatocellular carcinoma; RR = response rate.

**Table 2:** Studies with tumor dose outcome correlation in HCC with resin micropsheres.

Author and Year of Publication.	Lau 1994 [19]	Hermann 2018 [20]	Kao 2012 [43]	Strigari 2010 [44]	Allimant 2018 [45]
Nb patients/lesions	18/na	121/na	10/na	73/na	37/na
Lesion size (cm)	na	na	na	2.9	5
MAA or ^90^Y Based dosimetry	MAA based	MAA based	^90^Y SPECT/CT	^90^Y SPECT/CT	^90^Y PET
Response evaluation	WHO	RECIST1.1	RECIST1.1	EASL	mRECIST
TD parameter (Gy)/threshold TD (TTD)	Mean TD 120 Gy	Mean TD 100 Gy	Mean TD < 91 Gy	BED 110 Gy	AUDVH_T_ 61 Gy
RRs for TD ≥ TTD vs. TD < TTD	87.5% vs. 12%	na	100% vs. na	TCP of 73%	TCP of 76.5%
Prediction of response for TTD	na	na	na	na	se = 76.5% spe= 75%
OS for TD ≥ TTD vs. TD < TTD	55 w vs. 26.6 w *p* = 0.005	14.1 m vs. 6.1 m *p* = 0.0001	na	na	na

Nb = number, na = non available, TTD = threshold tumor dose, w = week, m = months, se = sensitivity; spe = specificity, acc = accuracy, BED = biological effective dose, TCP = tumor control probability, AUDVH_T_ = area under the tumor dose volume histogram; RR = response rate; OS = Overall Survival.

**Table 3:** Studies with NLD correlation with liver toxicity in HCC patients using resin and glass microspheres.

Author and Year of Publication	Strigari 2010 [44]	Allimant 2018 [45]	Garin 2013 [5]	Chiesa 2015 [48]	Garin 2017 [22]	Chan 2018 [51]
Nb of patients	73	37	71	52	85	35 (27 HCC, 7 metastasis)
Product	resin	resin	glass	glass	glass	glass
MAA- or ^90^Y-Based dosimetry	^90^Y SPECT/CT	^90^Y PET	MAA based	MAA based	MAA based	^90^Y PET
Toxicity evaluation	G ≥ 2	REILD	Clinically relevant, G ≥ 3 and permanent	Any liver decompensation	Clinically relevant, G ≥ 3 and permanent	G ≥ 2
NLD parameter/normal liver threshold dose (NLTD)	NPL BED 52 Gy	AUDVH_NPL_ na	NPLD 100 Gy + HR of <30% *p* = 0.032	WNLD 75 Gy	NPLD na	NPLD 54 Gy
NTCP for an NLD larger than an NLTD	50%	na	na	15%	na	50%
NLD parameters for patients with toxicity and no toxicity	na	78.9 Gy vs 53.8 Gy *p* = 0.04	na	na	104.7 Gy vs 79.5 Gy *p* = 0.028	na

Nb = number, na = not available, NLD = normal liver dose, NTCP = nontumor complication probability, BED = biologically effective dose, AUDVH_NPL_ = area under the normal perfused liver dose volume histogram, NPL = normal perfused liver, NPLD = normal perfused liver dose, HR = hepatic reserve, WNLD = whole normal liver dose.

## Abbreviations

A_I_	activity to inject
AUDVH	area under the dose volume histogram
BED	biologically effective dose
D	absorbed dose
DVH	dose volume histogram
D70	minimum dose provided to 70% of the volume of interest
HR	hepatic reserve
LSF	lung shunt fraction (total lung uptake/(total lung uptake + total liver uptake))
MIRD	medical internal radiation dose
NPLD	normal perfused liver dose
NTCP	nontumor complication probability
PLD	perfused liver dose
SIRT	selective internal radiation therapy
TCP	tumor control probability
TD	tumor dose
TTD	tumor threshold dose
TUR	tumor uptake ratio (total tumor uptake/total perfused liver uptake)
VOI	volume of interest
WLD	whole liver dose
WNPLD	whole normal perfused liver dose

## References

[1] Salem R., Lewandowski R.J., Mulcahy M.F., Riaz A., Ryu R.K., Ibrahim S., Atassi B., Baker T., Gates V., Miller F.H. (2010). Radioembolization for hepatocellular carcinoma using Yttrium-90 microspheres: A comprehensive report of long-term outcomes. Gastroenterology.

[2] Mazzaferro V., Sposito C., Bhoori S., Romito R., Chiesa C., Morosi C., Maccauro M., Marchiano A., Bongini M., Lanocita R. (2013). Yttrium-90 radioembolization for intermediate-advanced hepatocellular carcinoma: A phase 2 study. Hepatology.

[3] Salem R., Gordon A.C., Mouli S., Hickey R., Kallini J., Gabr A., Mulcahy M.F., Baker T., Abecassis M., Miller F.H. (2016). Y90 Radioembolization Significantly Prolongs Time to Progression Compared With Chemoembolization in Patients With Hepatocellular Carcinoma. Gastroenterology.

[4] Sangro B., Carpanese L., Cianni R., Golfieri R., Gasparini D., Ezziddin S., Paprottka P.M., Fiore F., Van B.M., Bilbao J.I. (2011). Survival after yttrium-90 resin microsphere radioembolization of hepatocellular carcinoma across Barcelona clinic liver cancer stages: A European evaluation. Hepatology.

[5] Garin E., Lenoir L., Edeline J., Laffont S., Mesbah H., Porée P., Sulpice L., Boudjema K., Mesbah M., Guillygomarc’h A. (2013). Boosted selective internal radiation therapy with 90Y-loaded glass microspheres (B-SIRT) for hepatocellular carcinoma patients: A new personalized promising concept. Eur. J. Nucl. Med. Mol. Imaging.

[6] Garin E., Rolland Y., Edeline J., Icard N., Lenoir L., Laffont S., Mesbah H., Breton M., Sulpice L., Boudjema K. (2015). Personalized dosimetry with intensification using 90Y-loaded glass microsphere radioembolization induces prolonged overall survival in hepatocellular carcinoma patients with portal vein thrombosis. J. Nucl. Med..

[7] Benson A.B., D’Angelica M.I., Abbott D.E., Abrams T.A., Alberts S.R., Anaya D.A., Anders R., Are C., Brown D., Chang D.T. (2019). Guidelines Insights: Hepatobiliary Cancers, Version 2.2019. J. Natl. Compr. Canc. Netw..

[8] Vogel A., Cervantes A., Chau I., Daniele B., Llovet J.M., Meyer T., Nault J.C., Neumann U., Ricke J., Sangro B. (2019). Hepatocellular carcinoma: ESMO Clinical Practice Guidelines for diagnosis, treatment and follow-up. Ann. Oncol..

[9] Vilgrain V., Pereira H., Assenat E., Guiu B., Ilonca A.D., Pageaux G.P., Sibert A., Bouattour M., Lebtahi R., Allaham W. (2017). Efficacy and safety of selective internal radiotherapy with yttrium-90 resin microspheres compared with sorafenib in locally advanced and inoperable hepatocellular carcinoma (SARAH): An open-label randomised controlled phase 3 trial. Lancet Oncol..

[10] Chow P.K.H., Gandhi M., Tan S.B., Khin M.W., Khasbazar A., Ong J., Choo S.P., Cheow P.C., Chotipanich C., Lim K. (2018). SIRveNIB: Selective Internal Radiation Therapy Versus Sorafenib in Asia-Pacific Patients With Hepatocellular Carcinoma. J. Clin. Oncol..

[11] Ricke J., Klumpen H.J., Amthauer H., Bargellini I., Bartenstein P., de Toni E.N., Gasbarrini A., Pech M., Peck-Radosavljevic M., Popovic P. (2019). Impact of combined selective internal radiation therapy and sorafenib on survival in advanced hepatocellular carcinoma. J. Hepatol..

[12] Garin E., Rolland Y., Campillo-Gimenez B., Edeline J. (2018). Negative phase 3 study of (90)Y microspheres versus sorafenib in HCC. Lancet Oncol..

[13] TheraSphere^TM^ Y-90 Glass Microspheres. https://www.bostonscientific.com/en-US/products/cancer-therapies/therasphere-y90-glass-microspheres.html.

[14] The Package Insert for SIR-Spheres^®^ Y-90 Resin Microspheres in English. https://www.sirtex.com/media/169278/pi-ec-13-spheres-ifu-eu-row.pdf.

[15] Chiesa C., Maccauro M., Romito R., Spreafico C., Pellizzari S., Negri A., Sposito C., Morosi C., Civelli E., Lanocita R. (2011). Need, feasibility and convenience of dosimetric treatment planning in liver selective internal radiation therapy with (90)Y microspheres: The experience of the National Tumor Institute of Milan. Q J. Nucl. Med. Mol. Imaging.

[16] Garin E., Rolland Y., Laffont S., Edeline J. (2016). Clinical impact of (99m)Tc-MAA SPECT/CT-based dosimetry in the radioembolization of liver malignancies with (90)Y-loaded microspheres. Eur. J. Nucl. Med. Mol. Imaging.

[17] Kao Y.H., Steinberg J.D., Tay Y.S., Lim G.K., Yan J., Townsend D.W., Budgeon C.A., Boucek J.A., Francis R.J., Cheo T.S. (2013). Post-radioembolization yttrium-90 PET/CT—Part 2: Dose-response and tumor predictive dosimetry for resin microspheres. EJNMMI Res..

[18] Walrand S., Hesse M., Chiesa C., Lhommel R., Jamar F. (2014). The low hepatic toxicity per Gray of 90Y glass microspheres is linked to their transport in the arterial tree favoring a nonuniform trapping as observed in posttherapy PET imaging. J. Nucl. Med..

[19] Lau W.Y., Leung W.T., Ho S., Leung N.W., Chan M., Lin J., Metreweli C., Johnson P., Li A.K. (1994). Treatment of inoperable hepatocellular carcinoma with intrahepatic arterial yttrium-90 microspheres: A phase I and II study. Br. J. Cancer.

[20] Hermann A.-L., Dieudonné A., Maxime R., Manuel S., Helena P., Gilles C., Laurent C., Rachida L., Vilgrain V., Group S.T. (2018). Role of 99mTc-Macroaggregated Albumin SPECT/CT based dosimetry in predicting survival and tumor response of patients with locally advanced and inoperable hepatocellular carcinoma (HCC) treated by selective intra-arterial radiation therapy (SIRT) with yttrium-90 resin microspheres, a cohort from SARAH study. J. Hepatol..

[21] Garin E., Lenoir L., Rolland Y., Edeline J., Mesbah H., Laffont S., Poree P., Clement B., Raoul J.L., Boucher E. (2012). Dosimetry based on 99mTc-macroaggregated albumin SPECT/CT accurately predicts tumor response and survival in hepatocellular carcinoma patients treated with 90Y-loaded glass microspheres: Preliminary results. J. Nucl. Med..

[22] Garin E., Rolland Y., Pracht M., Le S.S., Laffont S., Mesbah H., Haumont L.A., Lenoir L., Rohou T., Brun V. (2017). High impact of macroaggregated albumin-based tumour dose on response and overall survival in hepatocellular carcinoma patients treated with (90) Y-loaded glass microsphere radioembolization. Liver Int..

[23] Garin E., Rolland Y., Edeline J. (2019). (90)Y-Loaded Microsphere SIRT of HCC Patients With Portal Vein Thrombosis: High Clinical Impact of 99mTc-MAA SPECT/CT-Based Dosimetry. Semin. Nucl. Med..

[24] Garin E., Lenoir L., Rolland Y., Laffont S., Pracht M., Mesbah H., Poree P., Ardisson V., Bourguet P., Clement B. (2011). Effectiveness of quantitative MAA SPECT/CT for the definition of vascularized hepatic volume and dosimetric approach: Phantom validation and clinical preliminary results in patients with complex hepatic vascularization treated with yttrium-90-labeled microspheres. Nucl. Med. Commun..

[25] Haste P., Tann M., Persohn S., LaRoche T., Aaron V., Mauxion T., Chauhan N., Dreher M.R., Johnson M.S. (2017). Correlation of Technetium-99m Macroaggregated Albumin and Yttrium-90 Glass Microsphere Biodistribution in Hepatocellular Carcinoma: A Retrospective Review of Pretreatment Single Photon Emission CT and Posttreatment Positron Emission Tomography/CT. J. Vasc. Interv. Radiol..

[26] Garin é., Palard X. (2017). Is there a place for nuclear medicine in the radioembolization of liver tumors?. Médecine Nucléaire.

[27] Kafrouni M., Allimant C., Fourcade M., Vauclin S., Guiu B., Mariano-Goulart D., Ben B.F. (2019). Analysis of differences between (99m)Tc-MAA S. EJNMMI Res..

[28] Wondergem M., Smits M.L., Elschot M., de Jong H.W., Verkooijen H.M., Van Den Bosch M.A., Nijsen J.F., Lam M.G. (2013). 99mTc-macroaggregated albumin poorly predicts the intrahepatic distribution of 90Y resin microspheres in hepatic radioembolization. J. Nucl. Med..

[29] Salem R., Padia S.A., Lam M., Bell J., Chiesa C., Fowers K., Hamilton B., Herman J., Kappadath S.C., Leung T. (2019). Clinical and dosimetric considerations for Y90: Recommendations from an international multidisciplinary working group. Eur. J. Nucl. Med. Mol. Imaging.

[30] Leung W.T., Lau W.Y., Ho S.K., Chan M., Leung N.W., Lin J., Metreweli C., Johnson P.J., Li A.K. (1994). Measuring lung shunting in hepatocellular carcinoma with intrahepatic-arterial technetium-99m macroaggregated albumin. J. Nucl. Med..

[31] Elschot M., Nijsen J.F., Lam M.G., Smits M.L., Prince J.F., Viergever M.A., Van Den Bosch M.A., Zonnenberg B.A., de Jong H.W. (2014). ((9)(9)m)Tc-MAA overestimates the absorbed dose to the lungs in radioembolization: A quantitative evaluation in patients treated with (1)(6)(6)Ho-microspheres. Eur. J. Nucl. Med. Mol. Imaging.

[32] Ulrich G., Dudeck O., Furth C., Ruf J., Grosser O.S., Adolf D., Stiebler M., Ricke J., Amthauer H. (2013). Predictive value of intratumoral 99mTc-macroaggregated albumin uptake in patients with colorectal liver metastases scheduled for radioembolization with 90Y-microspheres. J. Nucl. Med..

[33] Ilhan H., Goritschan A., Paprottka P., Jakobs T.F., Fendler W.P., Todica A., Bartenstein P., Hacker M., Haug A.R. (2015). Predictive Value of 99mTc-MAA SPECT for 90Y-Labeled Resin Microsphere Distribution in Radioembolization of Primary and Secondary Hepatic Tumors. J. Nucl. Med..

[34] Kucuk O.N., Soydal C., Araz M., Ozkan E., Aras G. (2013). Evaluation of the response to selective internal radiation therapy in patients with hepatocellular cancer according to pretreatment (99m)Tc-MAA uptake. Clin. Nucl. Med..

[35] Gnesin S., Canetti L., Adib S., Cherbuin N., Silva M.M., Bize P., Denys A., Prior J.O., Baechler S., Boubaker A. (2016). Partition Model-Based 99mTc-MAA SPECT/CT Predictive Dosimetry Compared with 90Y TOF PET/CT Posttreatment Dosimetry in Radioembolization of Hepatocellular Carcinoma: A Quantitative Agreement Comparison. J. Nucl. Med..

[36] Jadoul A., Bernard C., Lovinfosse P., Gerard L., Lilet H., Cornet O., Hustinx R. (2020). Comparative dosimetry between (99m)Tc-MAA SPECT/CT and (90)Y PET/CT in primary and metastatic liver tumors. Eur. J. Nucl. Med. Mol. Imaging.

[37] Richetta E., Pasquino M., Poli M., Cutaia C., Valero C., Tabone M., Paradisi B.P., Pacilio M., Pellerito R.E., Stasi M. (2019). PET-CT post therapy dosimetry in radioembolization with resin (90)Y microspheres: Comparison with pre-treatment SPECT-CT (99m)Tc-MAA results. Phys. Med..

[38] Rhee S., Kim S., Cho J., Park J., Eo J.S., Park S., Lee E., Kim Y.H., Choe J.G. (2016). Semi-Quantitative Analysis of Post-Transarterial Radioembolization (90)Y Microsphere Positron Emission Tomography Combined with Computed Tomography (PET/CT) Images in Advanced Liver Malignancy: Comparison With (99m)Tc Macroaggregated Albumin (MAA) Single Photon Emission Computed Tomography (SPECT). Nucl. Med. Mol. Imaging.

[39] Kokabi N., Galt J.R., Xing M., Camacho J.C., Barron B.J., Schuster D.M., Kim H.S. (2014). A simple method for estimating dose delivered to hepatocellular carcinoma after yttrium-90 glass-based radioembolization therapy: Preliminary results of a proof of concept study. J. Vasc. Interv. Radiol..

[40] Ho C.L., Chen S., Cheung S.K., Leung Y.L., Cheng K.C., Wong K.N., Wong Y.H., Leung T.W.T. (2018). Radioembolization with (90)Y glass microspheres for hepatocellular carcinoma: Significance of pretreatment (11)C-acetate and (18)F-FDG PET/CT and posttreatment (90)Y PET/CT in individualized dose prescription. Eur. J. Nucl. Med. Mol. Imaging.

[41] Chan K.T., Alessio A.M., Johnson G.E., Vaidya S., Kwan S.W., Monsky W., Wilson A.E., Lewis D.H., Padia S.A. (2018). Prospective Trial Using Internal Pair-Production Positron Emission Tomography to Establish the Yttrium-90 Radioembolization Dose Required for Response of Hepatocellular Carcinoma. Int. J. Radiat. Oncol. Biol. Phys..

[42] Kappadath S.C., Mikell J., Balagopal A., Baladandayuthapani V., Kaseb A., Mahvash A. (2018). Hepatocellular Carcinoma Tumor Dose Response After (90)Y-radioembolization With Glass Microspheres Using (90)Y-SPECT/CT-Based Voxel Dosimetry. Int. J. Radiat. Oncol. Biol. Phys..

[43] Kao Y.H., Tan A.E.O., Burgmans M.C., Irani K.G., Khoo L.S., Lo R.H.G., Tay K.H., Tan B.S., Chow P.K.H., Ng D.C.E. (2012). Image-guided Personalized Predictive Dosimetry by Artery-Specific SPECT/CT Partition Modeling for Safe and Effective 90Y Radioembolization. J. Nucl. Med..

[44] Strigari L., Sciuto R., Rea S., Carpanese L., Pizzi G., Soriani A., Iaccarino G., Benassi M., Ettorre G.M., Maini C.L. (2010). Efficacy and toxicity related to treatment of hepatocellular carcinoma with 90Y-SIR spheres: Radiobiologic considerations. J. Nucl. Med..

[45] Allimant C., Kafrouni M., Delicque J., Ilonca D., Cassinotto C., Assenat E., Ursic-Bedoya J., Pageaux G.P., Mariano-Goulart D., Aho S. (2018). Tumor Targeting and Three-Dimensional Voxel-Based Dosimetry to Predict Tumor Response, Toxicity, and Survival after Yttrium-90 Resin Microsphere Radioembolization in Hepatocellular Carcinoma. J. Vasc. Interv. Radiol..

[46] Palard X., Edeline J., Rolland Y., Le S.S., Pracht M., Laffont S., Lenoir L., Boudjema K., Ugen T., Brun V. (2018). Dosimetric parameters predicting contralateral liver hypertrophy after unilobar radioembolization of hepatocellular carcinoma. Eur. J. Nucl. Med. Mol. Imaging.

[47] Sangro B., Gil-Alzugaray B., Rodriguez J., Sola I., Martinez-Cuesta A., Viudez A., Chopitea A., Inarrairaegui M., Arbizu J., Bilbao J.I. (2008). Liver disease induced by radioembolization of liver tumors: Description and possible risk factors. Cancer.

[48] Chiesa C., Mira M., Maccauro M., Spreafico C., Romito R., Morosi C., Camerini T., Carrara M., Pellizzari S., Negri A. (2015). Radioembolization of hepatocarcinoma with (90)Y glass microspheres: Development of an individualized treatment planning strategy based on dosimetry and radiobiology. Eur. J. Nucl. Med. Mol. Imaging.

[49] Dancey J.E., Shepherd F.A., Paul K., Sniderman K.W., Houle S., Gabrys J., Hendler A.L., Goin J.E. (2000). Treatment of nonresectable hepatocellular carcinoma with intrahepatic 90Y-microspheres. J. Nucl. Med..

[50] Shepherd F.A., Rotstein L.E., Houle S., Yip T.C., Paul K., Sniderman K.W. (1992). A phase I dose escalation trial of yttrium-90 microspheres in the treatment of primary hepatocellular carcinoma. Cancer.

[51] Chan K.T., Alessio A.M., Johnson G.E., Vaidya S., Kwan S.W., Monsky W., Wilson A.E., Lewis D.H., Padia S.A. (2018). Hepatotoxic Dose Thresholds by Positron-Emission Tomography After Yttrium-90 Radioembolization of Liver Tumors: A Prospective Single-Arm Observational Study. Cardiovasc Interv. Radiol..

[52] Spreafico C., Sposito C., Vaiani M., Cascella T., Bhoori S., Morosi C., Lanocita R., Romito R., Chiesa C., Maccauro M. (2018). Development of a prognostic score to predict response to Yttrium-90 radioembolization for hepatocellular carcinoma with portal vein invasion. J. Hepatol..

[53] Garin E., Tzelikas L., Guiu B., Chalaye J., Edeline J., De Baere T., Tacher V., Robert C., Assenat E., Terroir-Cassou-Mounat M. (2020). Major impact of personalized dosimetry using 90Y loaded glass microspheres SIRT in HCC: Final overall survival analysis of a multicenter randomized phase II study (DOSISPHERE-01). JCO.

[54] Lau W.Y., Kennedy A.S., Kim Y.H., Lai H.K., Lee R.C., Leung T.W., Liu C.S., Salem R., Sangro B., Shuter B. (2012). Patient selection and activity planning guide for selective internal radiotherapy with yttrium-90 resin microspheres. Int. J. Radiat. Oncol. Biol. Phys..

